# A reliable vaccine tracking and monitoring system for health clinics using blockchain

**DOI:** 10.1038/s41598-022-26029-w

**Published:** 2023-01-11

**Authors:** Kamanashis Biswas, Vallipuram Muthukkumarasamy, Guangdong Bai, Mohammad Jabed Morshed Chowdhury

**Affiliations:** 1 Australian Catholic University, Brisbane, Australia; 2 Griffith University, Gold Coast, Australia; 3 The University of Queensland, Brisbane, Australia; 4 La Trobe University, Melbourne, Australia

**Keywords:** Computer science, Information technology

## Abstract

Vaccines are delicate biological substances that gradually become inactive over time and must be kept under a recommended temperature range of 2–8 °C for both short and long-term storage. Exposure to heat or freezing temperatures can highly affect the immunological properties of these vaccines and make them completely ineffective. Research shows that vaccine exposure to temperatures outside the recommended range is 33% in developed countries and 37.1% in developing countries. In practice, vaccines are stored in refrigerators, while thermometers and data loggers are used to record and monitor temperatures. However, traditional systems are unreliable due to lack of battery backup, human error, periodic logging of temperatures, etc. Therefore, an effective and reliable vaccine tracking and monitoring system is urgently needed. This paper proposes a blockchain-based, smart contract enabled solution that ensures an enhanced level of security, transparency, and traceability of stored vaccines in a health clinic, and enables the complete history of every vaccine to be checked from the day the vaccine is received by the health clinic to the date it is used or expires. We also formally analyze the resiliency of the proposed system against several attacks and compare the system with existing blockchain and non-blockchain-based solutions.

## Introduction

Every year immunization protects millions of lives from many serious diseases. Over the past two decades, the immunization rate has increased significantly due to affordable cost and advancement in delivery systems and cold chain management technologies^1^. In the last three years, the world has seen global vaccine development efforts in response to the 2019 novel coronavirus (2019-nCoV) outbreak^2,3^ that resulted in several Covid vaccines as shown in Table 1. Some of these vaccines are freeze-sensitive and must be preserved within the recommended temperature during the whole lifetime. According to the supply annual report of the United Nations International Children’s Emergency Fund (UNICEF), the number of freeze-sensitive vaccines has increased by 50% since 2007^4^. However, standards for the maintenance, stock management, and distribution of vaccines remain a problem. For example, by 2020 only 29% of Global Alliance for Vaccines and Immunization (GAVI) eligible countries have met minimum temperature control standards, while only 10% of health facilities were found to be using adequate cold chain equipment^5^. According to the Vaccine Adverse Event Reporting System (VAERS) report, 23% of vaccination errors between 2000 and 2013 were caused by storage and dispensing issues. 88% of the reports recorded that vaccines kept outside of the recommended temperature range were administered to patients^6^. In practice, vaccines are stored in domestic, portable, and purpose-built vaccine refrigerators (PBVR) to keep them safe. However, attempts to address the issues of safe vaccine storage have not taken advantage of rapid developments in computing. Most of the existing solutions do not include battery backup systems and require manual readings or logging of temperatures, which can be subject to human tampering/error^7^. Further, temperature fluctuations in domestic refrigerators can be caused by the defrosting cycle in frost-free refrigerators or by opening the door. These necessitate the importance of a reliable vaccine monitoring and tracking system to detect changes in temperatures in real-time.

**Table 1:** Covid vaccine lifetime.

Vaccine lifetime	Pfizer BionTech		AstraZeneca		Moderna	
	Temperature	Duration	Temperature	Duration	Temperature	Duration
Long term storage	− 80 to − 60 °C	9 months	2 to 8 °C	6 months	− 25 to − 15 °C	7 months
Short term storage	2 to 8 °C	31 days	N/A	N/A	2 to 8 °C	30 days
Removing from the fridge	2 to 25 °C	2 h (prior to dilution)	2 to 25 °C	6 h	8 to 25 °C	24 h
Punctured	2 to 25 °C	6 h (after dilution)	2 to 25 °C	6 h	8 to 25 °C	6 h

In March 2021, several cold chain breaches were reported in Australia^8^. Moreover, a 2019 report found that thousands of patients of a Sydney clinic were asked to be re-vaccinated due to incorrect or out-of-date storage of vaccines since 2010^9^. A cold chain breach during transportation in Finland, left staff only an hour to prepare the doses and 6 h to find people to receive them^10^. A similar incident was reported by the staff of an East Sydney clinic which received 100 batches of AstraZeneca vaccines with the temperature device attached to the parcel showing that the vaccines were exposed to a higher temperature during transmission^11^. Therefore, every temperature violation event must be recorded and reported immediately to ensure the safety of vaccines.

Two other factors demonstrate the importance of the safe storage of vaccines in health premises. First, a growing number of cyberattacks have targeted critical infrastructures like hospitals and health clinics during the past few years^12^. For example, in December 2020, hackers compromised a European Medicines Agency (EMA) server and leaked Pfizer/BioNTech Covid-19 data onto the Internet^13^. Another cyberattack forced health clinics in the US and Italy to shut down their Covid-19 vaccination programs during the pandemic^14^. Second, the pandemic has accelerated the necessity of transparency and traceability in the vaccine supply chain^15^. People now want to know the full history of the vaccines that they are consuming. Although numerous Internet of Things (IoT) and blockchain-based solutions are proposed by researchers, these generic solutions mainly focus on the distribution and delivery of vaccines, not their storage.

To overcome these limitations, this paper proposes a smart contract enabled vaccine storage and monitoring system that records detailed information about every individual vaccine in an immutable and incorruptible decentralized database. Any deviation from the required standards (e.g., exposure to higher temperatures) will be immediately identified, reported and recorded in the blockchain. Thus, the system would allow patients to check whether the vaccine is safe before taking the shot. The contributions of this research are two-fold:

1. An architectural model of blockchain-based vaccine storage and monitoring system to ensure transparency, traceability, and real-time monitoring,
2. Implementation and formal verification of security properties to ensure safety and correctness of the proposed system.

The rest of the paper is organized as follows: “Background and related works” section provides the background and related works. “Issues in a cold chain breach management system” section identifies the issues in a cold chain breach management system. “System design” section demonstrates the proposed blockchain-based vaccine monitoring and tracking system. “Security analysis” section presents a security analysis of the proposed system. Finally, “Conclusion” section concludes the paper.

**Table 2:** Comparison with existing industry solutions.

Characteristics	OneTemp^21^	EasyLog^22^	MONNIT^23^	Sensoscientific^24^	TempDefender G2^25^	Proposed system
Technology	Bluetooth	USB/WiFi	WiFi	WiFi	Wired	WiFi
Data storage	Centralised	Centralised	Centralised	Centralised	Local	Distributed
Integrity	No	No	No	Yes	No	Yes
Traceability	Yes	No	Yes	Yes	No	Yes
Scalability	No	No	No	No	No	Yes
Real-time alert	Yes	No	Yes	Yes	No	Yes
Security	No	No	No	No	No	Yes
Access control	No	No	No	No	No	Yes

## Background and related works

Vaccines are categorized into four different groups based on several factors such as stability, liveliness, heat, and freeze-sensitivity. Live vaccines use weakened, attenuated versions of the germ that can replicate^16^. These vaccines require careful maintenance of the vaccine cold chain. On the other hand, inactivated vaccines contain the killed versions of the germ and thus they are non-replicating. Similarly, subunit vaccines are non-replicating vaccines that use only specific pieces of the germ such as protein, sugar or capsid. Both these two latter categories of vaccines are typically available in liquid form and are generally more stable. However, these vaccines can be freeze-sensitive and must be stored and distributed within the recommended range of temperature. Unlike these, toxoid vaccines contain a toxin that creates immunity to the parts of the germ that causes a disease. This type of vaccine remains stable at elevated temperatures, even for long periods of storage. However, for any kind of vaccine, it is crucial to have a reliable system that will maintain the recommended temperatures from the manufacturer to the point of use.

Although the World Health Organisation (WHO) urges the need for an end-to-end temperature monitoring system in the cold chain, there is a long history of breaches in temperature during vaccine storage and distribution. Dipika et al. found that 14% to 35% of refrigerators or transport shipments exposed vaccines to freezing temperatures^17^. Since more expensive, freeze-sensitive vaccines are being introduced into immunization schedules, freeze prevention is very critical to ensure that people are receiving fully potent vaccines.

Fatima et al. propose a model for cold chain monitoring using a Colored Petri Net (CPN) which focuses mainly on the vaccine warehouse storage process^18^. Although the authors claim that their proposed model can reduce the risks of cold chain breach and monitor temperatures in real-time, the paper does not indicate how a breach will be detected and notified to the corresponding authority.

A data-centric and Internet of Things (IoT) based cold chain monitoring system is proposed in^19^ by Hasnat et al. The system focuses on real-time data acquisition and monitoring of the temperature and humidity of the carrier during vaccine distribution and transportation processes. This mobile app-based supervision system ensures transparency and efficiency in the whole process. However, the integrity and security of critical information are not addressed in this paper.

In^20^, the authors proposed a methodology for cold storage monitoring and tracking using a LoRaWAN (LoRa Wide Area Network) gateway, a LORIOT network server, a user application and an end node to monitor the temperature, pressure, and humidity data collected by the built-in sensors. Although the system provides security against the unauthorized removal of sensors, it fails to ensure data provenance and transparency.

In addition to the above mechanisms, there are numerous IoT based solutions designed and developed by the industry to monitor and track vaccines in real-time^21–25^. However, these solutions have several limitations such as a single point of failure (centralized system), limited communication range, integrity and scalability issues, and lack of transparency. Table 2 presents a comparison between our proposed system with several industry solutions.

Unlike IoT based solutions, Yong et al. proposed an in- telligent vaccine supply and supervision system based on blockchain and machine learning technologies to overcome the problems of vaccine expiration and record fraud^26^. The proposed system deploys a smart contract to detect expiry dates and retrieve query information about vaccines. However, the authors have not considered the security aspects or the need for an appropriate access control mechanism. Moreover, it does not include all stakeholders involved in the vaccine delivery and distribution processes.

Musamih et al.^27^ presented an Ethereum blockchain-based solution to automate the traceability of Covid-19 vaccines to ensure data provenance, transparency, security, and accountability of the vaccine supply chain. They also analyzed the resilience of the proposed system against Man-in-the-middle (MITM) attacks. However, the solution focuses on manufacturing and distribution processes, not storage and real-time monitoring in a health clinic.

Another blockchain-based generic scheme, VaCoChain, pro- posed by Verma et al. fuses blockchain, unmanned aerial vehicles (UAVs) and fifth-generation (5G) communication services for timely vaccine distribution during pandemics^28^. However, its primary focus is the timely delivery of the vaccine using UAVs, not the traceability of the stored information.

As above, most of the blockchain-based solutions are proposed to ensure transparency and traceability in the vaccine supply chain^29–32^. However, there is an urgent need to develop a distributed and secure vaccine storage and tracking system for health facilities since they are more vulnerable to security attacks nowadays. Therefore, this research (based on our initial work^33^) proposes such a system.

## Issues in a cold chain breach management system

This section provides details of a cold chain breach management system and presents an attack scenario to identify potential security issues. Some of these can have significant impacts on the immunization system and can be detrimental to human health.

### Cold chain breach management

**Figure 1:**
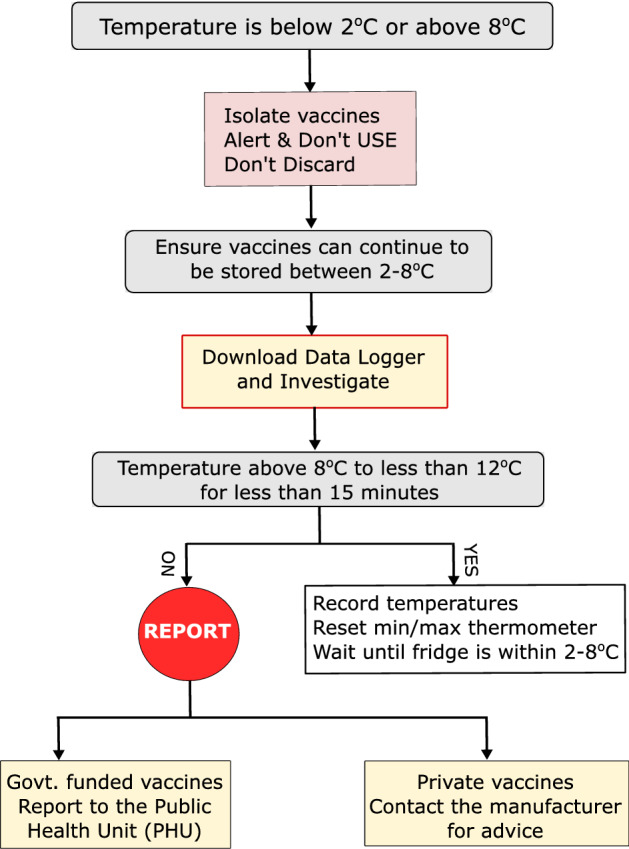
Managing a cold chain breach.

Most of the existing systems for managing a cold chain breach involve human interventions and are thus subject to manipulation. Figure 1 presents the cold chain breach management protocol implemented by New South Wales (NSW) Health, Australia^34^. In this process, all vaccines must be stored within the recommended temperature range (2–8 °C) in PBVRs at all times. To ensure the recommended temperature range, a data logger and a minimum/maximum thermometer with a digital display are used to manually monitor the temperature. The current, minimum, and maximum temperatures of the refrigerator are recorded every day at the opening and closing of practice, or once a week for practices not opened daily, and prior to using vaccines. It is also mandatory for every health practice to perform a self-audit annually that includes servicing vaccine refrigerators, calibrating thermometers or data loggers, and changing batteries.

If a cold chain breach occurs during the storage of vaccines in a health clinic, then the vaccines need to be transferred to an alternate purpose-built refrigerator or cooler (if available) and labeled with a ‘*Don’t USE*’ sign. The staff who manages or administers vaccines must download and review the data logging report to assess whether the temperature has been between 8 and 12 °C for more than 15 minutes. If the duration of the breach and temperature of the refrigerator reached this threshold, the incident has to be reported either to Public Health Unit (PHU) for Government funded vaccines or to manufacturers (for private vaccines). The health clinic also needs to complete and return the ‘*cold chain breach reporting form*’ to the local PHU for their advice. This system has a number of limitations as follows:

- *Malfunctioning hardware* The system mainly relies on a min/max thermometer. If this thermometer is malfunctioning due to mechanical faults, all vaccines stored in a particular PBVR may need to be discarded.
- *Human error* Since the temperature is recorded manually twice a day, staff might forget to record the refrigerator temperature and enter some arbitrary, within the range, figures.
- *Rogue health clinic* A rogue health clinic can easily manipulate the data, to protect its reputation.
- *Outsider attack* Most health clinics do not implement standard security mechanisms and thus can be an easy target for attackers, who could manipulate or inject false information into the system.

**Figure 2:**
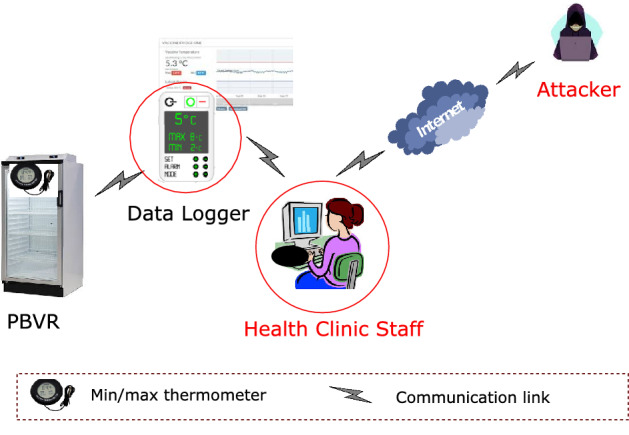
An external attack scenario.

### An attack scenario

Figure 2 presents an attack scenario where an attacker attempts to compromise the health clinic computer connected to the data logger. Since health clinic computers are connected to the Internet, a single security hole in the system will allow the attackers to compromise the whole system and manipulate stored information. In the worst-case scenario, the attackers may penetrate the data logger and can inject malicious codes into the logger. Our proposed blockchain-based vaccine storage and monitoring system can effectively prevent external attacks and preserve data integrity. Here we explain how the proposed system will overcome these pitfalls.

- *Malfunctioning hardware* The proposed system uses a number of temperature sensors in addition to a min/- max thermometer that periodically sends data to the aggregators. By checking the received information, the aggregators can identify if any temperature sensor or the min/max thermometer is malfunctioning.
- *Human error* The proposed system has very minimal human interaction as most of the processes are written on smart contracts and thus executed whenever a certain condition is met. For example, the temperature monitor- ing and review process is completely automated and thus there is no need for human intervention in this process. This minimizes human errors as well as ensures data integrity since health clinic staff would not be able to change any recorded information.
- *Rogue health clinic* Since blockchain stores information in an append-only format and all blocks form a hash chain, it is not possible for a health clinic to alter any information once recorded in the chain. In addition to health clinics, regulatory bodies are included in the proposed system. If a cold chain breach takes place, regulatory bodies will also be notified immediately.
- *External attacks* The scope for external attacks is very limited in the proposed system for several reasons. First, we use a consortium blockchain where only authorized entities can participate in the transactions. Second, to inject a block with false information, an attacker must compromise more than 50% of the pre-selected miners (i.e., verifiers of a block) which is really difficult. Third, if attackers compromise a single blockchain node, they won’t be able to alter any recorded information in the blockchain.

The proposed system can also be used with any type of refrigerator. Although it is recommended to store vaccines in PBVRs in Australia, many developing countries use domestic refrigerators since PBVRs are very expensive.

## System design

**Figure 3:**
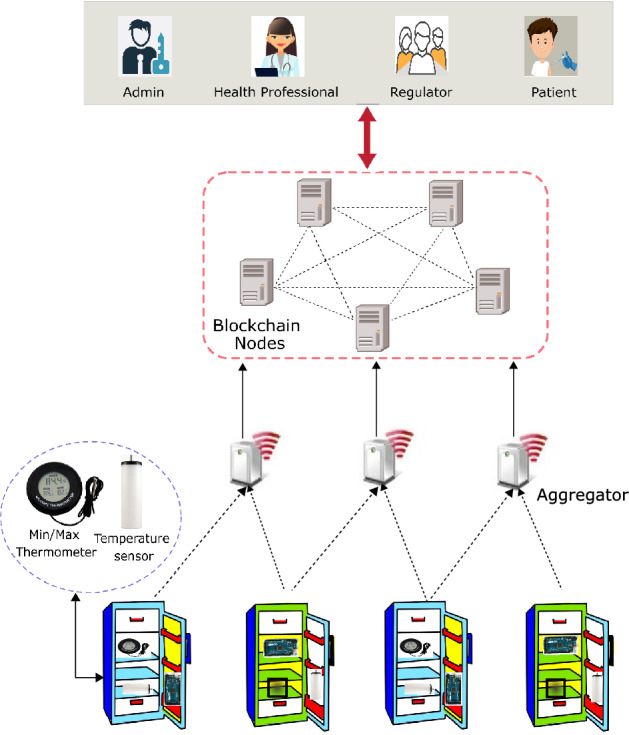
The proposed system.

This section first briefly describes the system components and then presents the architectural model of the proposed system. Figure 3 shows an overview of the proposed vaccine tracking and monitoring system.

### System components

The system components described below play significant roles in tracking and monitoring vaccines in real-time.

#### Sensory devices

As mentioned earlier, vaccines can be stored in PBVRs, domestic, and portable refrigerators. Domestic refrigerators are subject to temperature fluctuations and therefore, we need a very accurate and efficient mechanism to monitor storage temperature. In the proposed model, we use several temperature sensors and a min/max thermometer attached to each refrigerator. We recommend using a min/max thermometer for the central refrigerator rack while the other racks should be occupied with a temperature sensor. These hot-swappable sensory devices will continuously send temperature readings to the aggregators.

#### Aggregator

The aggregators continuously receive temperature readings from the sensory devices, aggregate them and periodically send these records to the blockchain nodes to reduce the storage and network overheads. For example, an aggregator can send aggregated readings to the upper-layer nodes after every 30 seconds. However, if it receives a temperature reading which is below or above the recommended range, it will send the information immediately. Another functionality of the aggregator is that it would also detect faulty sensors and pass that information to the blockchain nodes. The aggregator can be programmed in such a way that it can monitor the behaviors of the sensory devices and detect faulty devices. Thus, the aggregators send two types of information to the blockchain nodes: (i) *regular messages* that include refrigerator id, temperature, and time, and (ii) *alert messages* that include either refrigerator id, temperature, time, and alert message (a breach in temperature) or refrigerator id, sensory device id, time, and alert message (faulty device).

**Figure 4:**
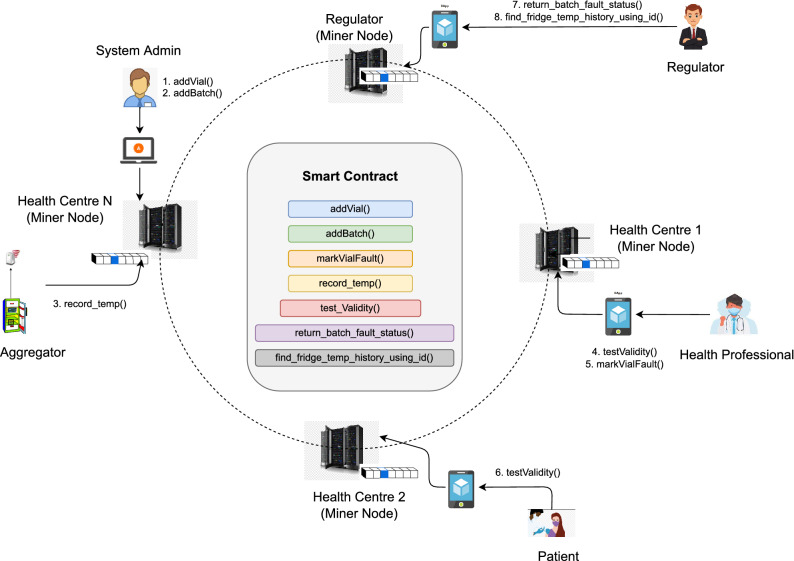
The architectural model of the proposed system.

#### Blockchain nodes

This layer consists of a number of blockchain nodes deployed by the health clinics. These nodes can be distributed geographically, and any health clinic would be able to join this network by simply deploying a blockchain node and connecting this node with the internal networks. We assume that both blockchain nodes and aggregators are equipped with Trusted Platform Modules (TPM) and thus can securely store sensitive information like secret keys. In our proposed model, all information exchanged between aggregators and blockchain nodes is encrypted using a symmetric key algorithm to preserve the confidentiality of the transmitted information. The working procedure of the blockchain layer is as follows. Blockchain nodes implement a smart contract that acts in different ways based on the received information. For example, if a blockchain node receives a regular message, the smart contract will be triggered, and the transaction will be verified by the miners and added to the blocks. On the other hand, if it receives an alert message, the message will be sent to all corresponding parties for immediate action in addition to adding the information to the chain.

#### Participants

The different groups of participants in the proposed system would have different functionalities and different levels of access to the system, as follows. Further details are provided in “The architectural model”.

- **Admin** Health clinic admins would have ‘*write access*’ to the chain and are responsible for storing all information related to vaccines (including discarded vaccines) in the chain.
- **Health professional** Health professionals such as doctors and nurses also have ‘*write access*’. They check the history of the vial before it is administered, can update the status of a vial once it is consumed, and also decide whether a particular batch of vials needs to be discarded.
- **Regulator** Regulators are employed by the state or federal government and can be part of the proposed system by simply deploying a blockchain node. This will enable them to be notified about any cold chain breach and follow up on the actions taken by the health clinic. Furthermore, regulators will also have a real picture of vaccine consumption to help them to take early actions to prevent vaccine shortages in times of peak demand.
- **Patients** Patients have only ‘*read access*’ to the proposed system. They can use a mobile app or the health clinic website, to download a snapshot of the vial history including all key information about the vial.

### The architectural model

Figure 4 presents the architectural model which shows the interactions among the entities in our proposed system. The core of this model is a smart contract that controls the data flow and stores summary information on the blockchain. However, the detailed information is stored in traditional file systems such as off-chain storage, because, for example, the amount of data generated by the sensors recording and transmitting temperature data periodically, is quite large. The proposed system logs the temperature data in the local system and uploads/sends only summary data (for example, the mean temperature of each fridge every 10 min) to the blockchain. In addition, the system captures and stores any events such as missing data readings or temperature readings being out of expected thresholds. In such situations, the system will create an alert message and notify corresponding entities. Since the event is recorded in the blockchain, the corresponding participant (e.g., regulators) can view this event at any time. To deploy the proposed system, the architectural model implements a consortium-based blockchain system, where we can control who can join the system and how. In addition, there is no need for any high computation-intensive consensus mechanisms which ensures scalability and improved performance of the system.
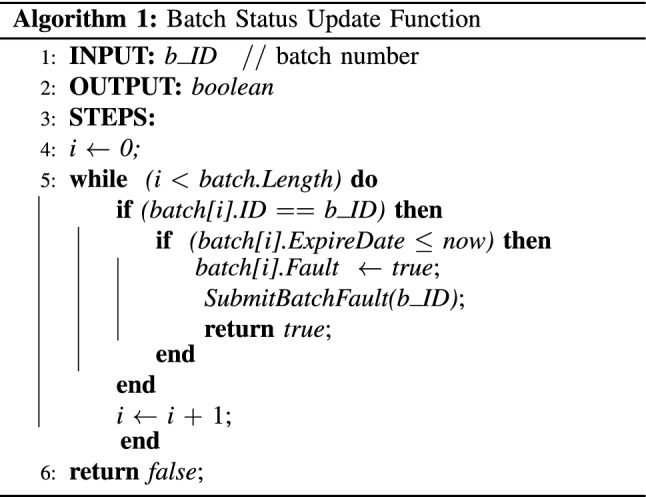


In the proposed system, healthcare clinics run the miner nodes and maintain the system. A special miner node is maintained by the regulator, which ensures that the regulator can always get access to the data stored by the health clinics. If any incident happens, they can check the validity of a vial or a batch of vials by invoking *return_batch_fault_status()* function in the smart contract. Similarly, health professionals can invoke *test_validity()* function to check the validity of the vials before applying them to the patient. If the function returns *false* but the records show that the vial has been exposed to the temperature for a short duration (less than 15 minutes), health professionals can still mark the vial as not safe to use and ask the health admin to discard the whole batch. In this situation, they will invoke *markVialFault()* function to register the whole batch as a faulty batch. Finally, the patient can also check the validity of the vial before it is administered to her body by invoking *test_validity()* function. The patient would be able to see all records pertaining to the vials from the date of registration by the health clinic to the date of consumption. Algorithm 1 presents the ‘*batch status update’* function used to check the expiry date of batches and update the status accordingly. Similarly, the ‘*batch status check*’ function presented in Algorithm 2 returns the current batch status when invoked by a health professional or health admin. The next subsection describes the interactions among different actors in the proposed system.
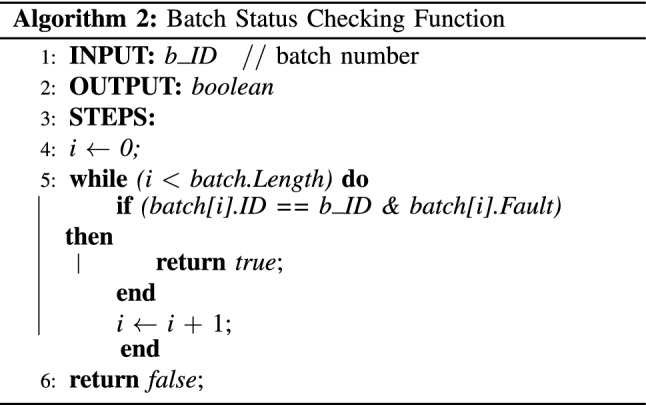


#### Workflow of the proposed system

**Figure 5:**
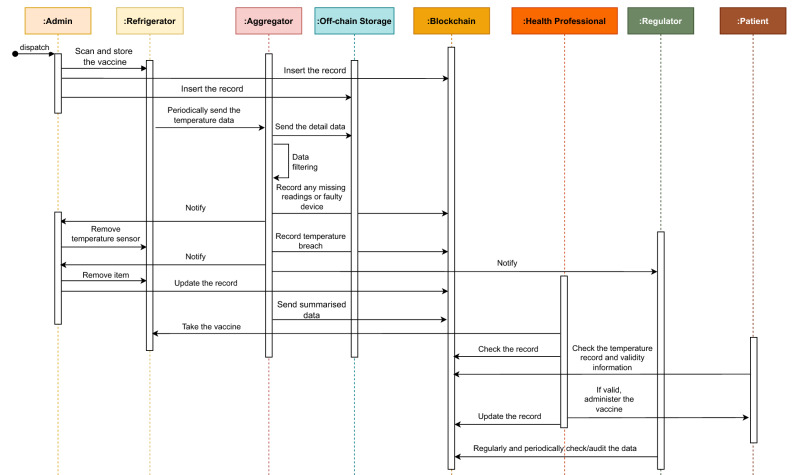
Sequence diagram of the proposed system.

Figure 5 presents the sequence diagram that provides a comprehensive view of the interactions among different entities. For the sake of simplicity, the workflow is described from the viewpoints of four actors: Admin, Regulator, Health Professional, and Patient.

*Role of admin:* Upon the arrival of vaccines at health clinics, the health admin stores all vaccines in the refrigerators and up-dates the records on the blockchain, including the arrival date, manufacturer details, batch number, vial range, refrigerator ID, storing temperature, expiry date, and comments. Now, the sensory devices in the refrigerators record the temperature and periodically send collected data to the aggregators. The aggregators aggregate received information and forward them to the off-chain module if no unusual event is detected. The off-chain module also regularly sends the summarized data to the blockchain. However, if an unusual event such as faulty device detection or a breach in temperature happens, the aggregators will carry out the following steps. If a sensory device is malfunctioning (e.g., sending ambiguous data), the aggregators will notify the health admin so that the health admin can remove or replace the faulty device. Similarly, if a breach in temperature is identified, an alert message will be sent to both the health admin and the regulators. This will enable the health admin to take appropriate actions to ensure vaccine safety and the regulators to follow up on the incident as needed. The health admin is also responsible for updating records if a batch is expired or discarded due to a cold chain breach. It is assumed that each vial could be uniquely identified by its batch number and refrigerator ID or using a unique identifier provided by the vaccine manufacturer.

*Role of regulator* Regulators can audit the recorded information from time to time since they have full access to the blockchain data. They will also receive a notification of temperature violations and thus, can keep track of such incidents for further investigation.

*Role of health professionals* Health professionals check the validity of a vaccine before administering it to the patient. In addition to adding the records of a consumed vaccine on the blockchain, they can discard a vaccine and update the information if they find it unsafe for use due to exposure to temperature (even if the vaccines are exposed to the incorrect temperature multiple times for a short period).

*Role of patients* Finally, patients can check the vaccine status and temperature records via a mobile app or health clinic website before taking the shot. This will ensure that they have received effective and safe vaccines.

### Implementation

The proposed system has been implemented on the Ethereum blockchain platform, and deployed and tested in a private Ethereum network. We have used *Solidity* to write the smart contract code and for the front-end, we have used the C# programming language.

**Figure 6:**
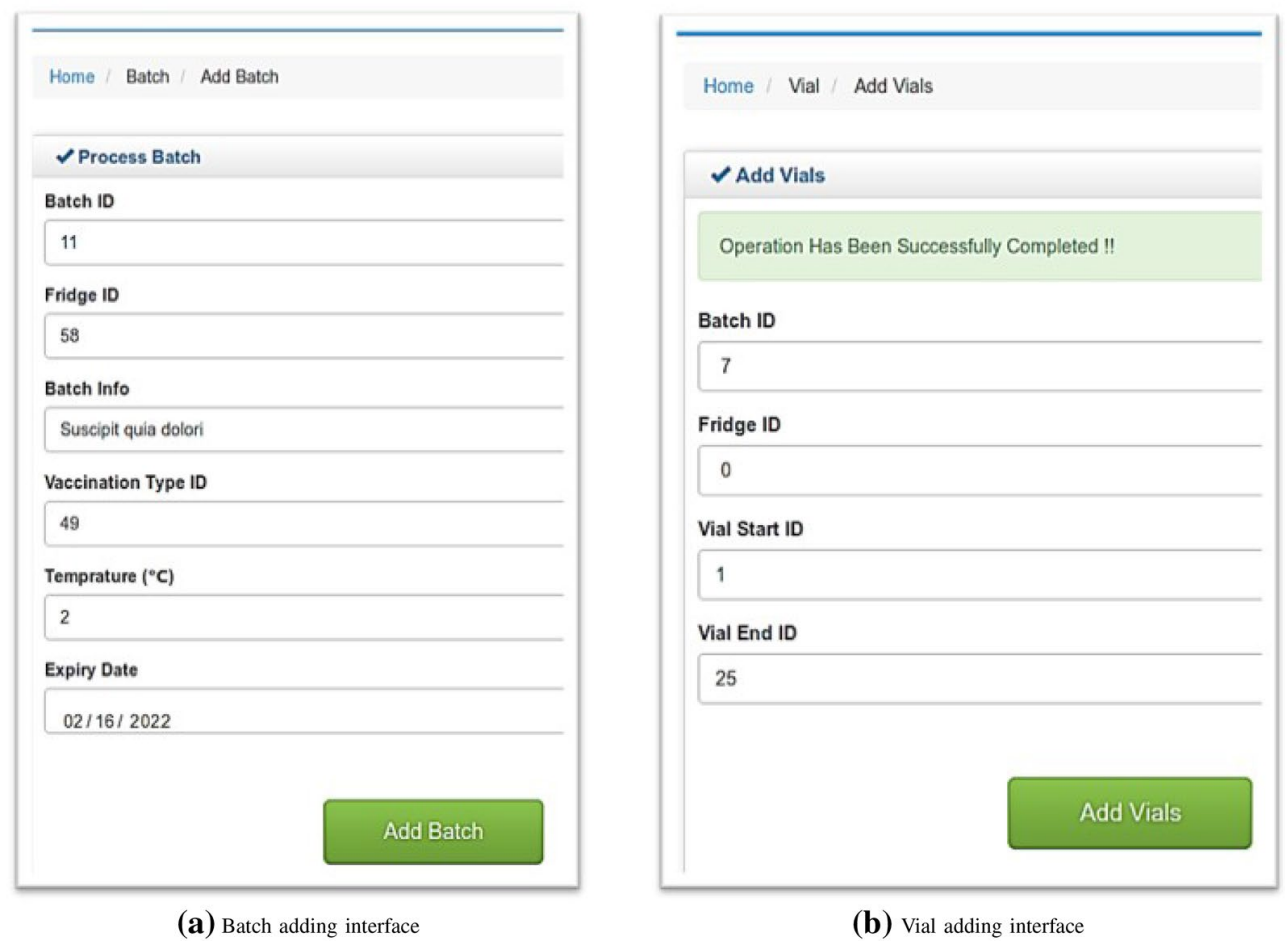
Adding batch and vial information in the blockchain.

**Figure 7:**
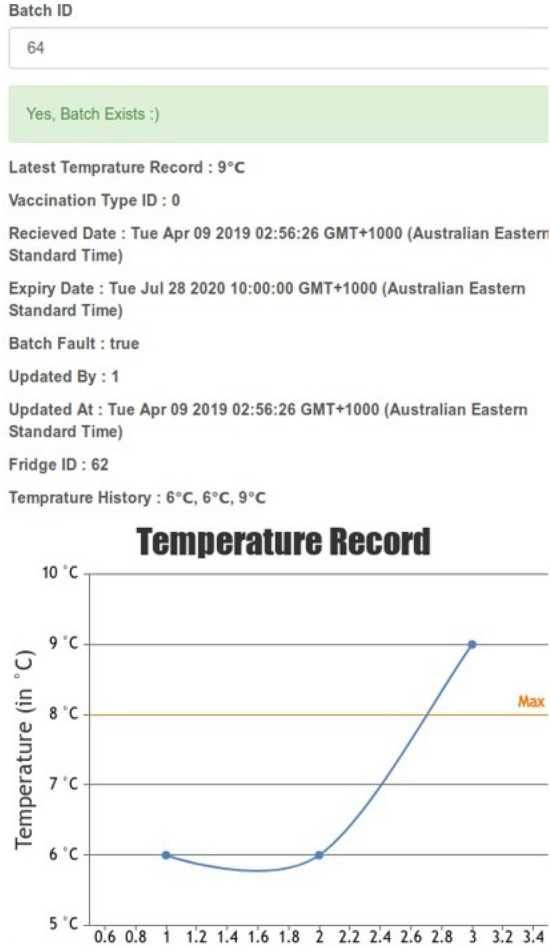
Temperature record check using batch id.

Figure 6 shows the interface to add batch and vial information to the blockchain. Health admins are responsible for adding vaccine information for their corresponding health clinics. They can enter the batch information (e.g., Batch ID, Fridge ID, Batch Info, Vaccine Type, Initial Temperature and Expiry Date) for a batch of vials in the blockchain. If they need to add any additional vaccine to an existing batch, they need to use the “Add Vials” interface as shown in Fig. 6b. Once the vial information is entered into the system and vaccines are placed in the refrigerators, the aggregator will collect and start sending the summary information for the newly added vials and batches to the blockchain nodes. Figure 7 shows the output of the batch status check for batch ID 64. It can be seen that the recorded temperature is not within the recommended range and therefore, the batch fault status is true.

### Comparative analysis

The implementation outcomes demonstrate that the proposed smart contract enabled monitoring and tracking system effectively identifies temperature violations and notifies the events immediately to the corresponding entities. In this section, we present a comparative analysis of the proposed system with different blockchain-based solutions. Table 3 shows that only the proposed system tracks and monitors vaccines stored in health premises, and that only the proposed system satisfies all required safety and reliability requirements.

## Security analysis

### System modelling

We use CSP#^35^ to model the proposed system, and then verify whether our design model satisfies the desired properties using PAT^36^ model checker. We chose CSP# because it integrates the features of the high-level modelling language (CSP) with a low-level procedural programming language (C#). This allows us to model the complex data structures which are extensively used to represent the properties and behaviors of the blockchain.

We define the system as a set of actors where each actor executes a sequence of processes in parallel. In particular, it is modelled as follows: $AllActors()=\,Manufacturer()\,\,\Vert |\,\, HealthClinic()\,\,\Vert |\,\,PublicUser();$ where $P \,\Vert |\, Q$ represents that two asynchronous processes *P* and *Q* running in parallel.

The process $Manufacturer(\,)$ is defined as follow: $[batchCount \,\, < MAXBATCHES]manufAddBatch\,\, \rightarrow \,\, AddBatch(permManufact)$;

$Manufacturer()\,\,\sqcap \,\,[batchCount\,\,>\,\,0] manufInvalBatch\,\,\rightarrow \,\, InvalidateBatch(permManufact); Manufacturer()$; where $[\,\,]$ represents the guard condition, $P\sqcap Q$ represents internal choice, and *P* and *Q* represent processes executed in sequence. This process models the manufacturer’s behaviors, which include adding vaccines in a batch and invalidating a batch of vaccines. The model of *AddBatch* is defined below.
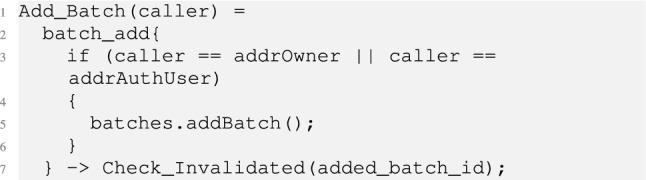


It first checks the caller identity (line 3), and then calls into a C# library which implements the batch and its management (line 5). The *Batch* is constructed as below.
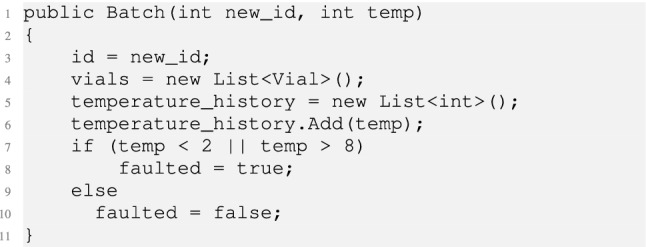


The *AddBatch* is then modelled as follows.
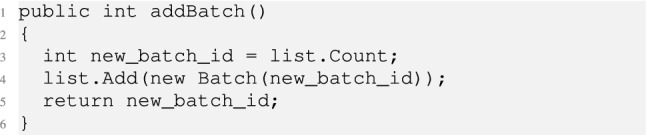

**Table 3:** Comparison with existing blockchain-based systems.

Characteristics	Yong et al.^26^	Musamih et al.^27^	VaCoChain^28^	Proposed system
Scope	Manufacturing and distribution	Manufacturing and distribution	Distribution	Storage and tracking
Platform	Ethereum	Ethereum	Ethereum	Ethereum
Blockchain type	Public permissioned	Public permissioned	Consortium	Consortium
Off-chain data storage	No	Yes	Yes	Yes
Traceability	Yes	Yes	Yes	Yes
Real-time monitoring	No	Yes	No	Yes
Formal security analysis	No	No	No	Yes

The process *HealthClinic*() models the behaviors of the clinic. The core behaviors of the clinic include recording consumption information when it consumes a vaccine and invalidating a vaccine batch if it detects a breach in the cold chain. The clinic can also add the patient information into the system and access the information about the vaccine.

The process of *PublicUser*() models the behaviors of a client, which includes reading the information about the vaccine, and looking up the history of a particular vaccine.

### Modelled attacks

In our formal analysis, we focus mainly on the integrity attack. For this category of attack, we explore whether some crucial information about the vaccine can be changed by the attacker. For example, a rival health clinic can attempt to invalidate every batch of vaccines, or an internal staff may attempt to change a vaccine’s status from invalid to valid. We exclude other attacks such as attacks on confidentiality from our attack model. Since the proposed system uses TPM and symmetric key encryption mechanisms to protect secret keys and information, we assume that the system can successfully defend itself against confidentiality attacks.

The behaviors of the attackers are embedded into the system model with a set of processes running in parallel. For example, a *RivalClinic* process forked from *HealthClinic*() can be added to model a health clinic attempting to sabotage all vaccines in the system. In addition, there is a process $UpdateBatchTemperature(callerPermission) \,\,\Vert |\,\, UpdateBatchTempValid(callerPermission, ID)\,\, \Vert |\,\, UpdateBatchTempInvalid(callerPermission, ID)$ to model the reaction to a temperature change in the environment. It includes three processes that are used to simulate batch temperature update behavior. *UpdateBatchTemperature* simply checks caller permission and the temperature readings. Depending on the temperature result, the batch ID is either sent to *UpdateBatchTempValid* or *UpdateBatchTempInvalid*, where the temperature update and corresponding events are logged. These processes are allowed only when the caller has *Fridge* permissions.

### Checked properties

For the desired properties, we define a set of assertions to capture them. The following set of properties is analyzed in this subsection.

- *P0: Deadlock-freeness*. A model is deadlock-free if, at any point in time, no node in the system enters a waiting state due to a resource being held by another waiting node. Deadlock-freeness is a readily checkable property by PAT.
- *P1: Ability to add batches*. The system should be functional so that batches can be added at any time.
- *P2: Ability to invalidate all*. The system should be functional such that once the vaccine becomes invalid, it must be invalidated in the system.
- *P3: Ability to detect temperature fault*. The system should be functional so that the temperature fault can immediately be detected.
- *P4: Non-missing of temperature fault*. The system should be functional and all temperature faults must be recorded on the blockchain.
- *P5: Resistance to patient data modification*. The system should be resistant to patient data integrity violations.
- *P6: Resistance to temperature history modification*. The system should be resistant to temperature history integrity violations.
- *P7: Resistance to batch fault revert*. The system should always release batch faults that cannot be reverted.
- *P8: Resistance to internal attacks*. This property checks whether an internal staff can change the vaccine status from invalid to valid.

### Results

In our model checking, we use different settings regarding the number of vials, batches, and patients, to simulate real-world scenarios. Table 4 lists the satisfiability of the checked properties, showing that all desired properties are satisfied. This implies that the proposed system protects the health clinic from all internal and external attacks that we identify in “Issues in a cold chain breach management system”. As an example, the proposed system actively defends against internal attacks (P8). Since all records are stored in the distributed ledger, it is impossible for individual staff to change the vaccine status. Similarly, to append a malicious block in the chain, the attackers need to take control of more than half of the miner nodes which is almost impossible. In addition to this, we also report the statistics regarding our experiments in Table 5. In general, the number of explored states increases as the problem domain becomes more complex. Most of the properties take the model checker for a comprehensive exploration of the state space, such that the number of explored states is too huge for manual analysis. This suggests the necessity of automatic analysis in verifying complex systems.

**Table 4:** Satisfiability of the checked properties.

Property	Experiment setting (#vials, #batches, #patients)			
	(2, 1, 1)	(2, 1, 3)	(3, 1, 5)	(5, 3, 5)
P0	Y	Y	Y	Y
P1	Y	Y	Y	Y
P2	Y	Y	Y	Y
P3	Y	Y	Y	Y
P4	Y	Y	Y	Y
P5	Y	Y	Y	Y
P6	Y	Y	Y	Y
P7	Y	Y	Y	Y
P8	Y	Y	Y	Y

**Table 5:** Number of states visited in verifying each property.

Property	Experiment setting (#vials, #batches, #patients)			
	(2, 1, 1)	(2, 1, 3)	(3, 1, 5)	(5, 3, 5)
P0	2288	8358	13,918	415,807
P1	43	46	46	46
P2	349	367	367	534
P3	43	46	46	46
P4	2288	8358	13,918	415,807
P5	2288	8358	13,918	415,807
P6	2288	8358	13,918	415,807
P7	9	9	9	9
P8	99	355	819	12,502

## Conclusion

This paper presents a blockchain-based vaccine monitoring and tracking system for health clinics which provides a traceable and useful audit trail of collected information for regulators^37^. The proposed system is secure and scalable since any new health clinic can join the system at any time. In addition to the inherent properties of blockchain, regulatory bodies also play a key role in the transparency of the system since any abnormal event will be notified immediately to the regulators. The outcomes of security analysis also show that the proposed system can successfully defend itself against a wide variety of attacks. One of the limitations of this work is that the proposed solution only focuses on in-house vaccine monitoring and tracking. Our future work will aim to design a blockchain-based system for the entire supply chain and compare it with other blockchain-based supply chain solutions. We will also investigate the impacts of autonomous intelligence and cloud computing in designing an effective vaccine supply chain management system.

## Data Availability

All data generated or analysed during this study are included in this published article and its supplementary information files (https://github.com/jabedongit/VacciChain).
